# Effects of ozone exposure on lipid metabolism in Huh-7 human hepatoma cells

**DOI:** 10.3389/fpubh.2023.1222762

**Published:** 2023-07-13

**Authors:** Jianhao Peng, Siyuan Wang, Yunlong Wang, Wanchao Yu, Yejun Zha, Shuxin Gao

**Affiliations:** ^1^College of Animal Science and Technology, Inner Mongolia University for Nationalities, Tongliao, China; ^2^Department of Orthopedic Trauma, Beijing Jishuitan Hospital, Beijing, China

**Keywords:** O_3_, Huh-7, lipid metabolism, oxidative stress, ROS

## Abstract

Ozone pollution is a major environmental concern. According to recent epidemiological studies, ozone exposure increases the risk of metabolic liver disease. However, studies on the mechanisms underlying the effects of ozone exposure on hepatic oxidative damage, lipid synthesis, and catabolism are limited. In this study, Huh-7 human hepatocellular carcinoma cells were randomly divided into five groups and exposed to 200 ppb O_3_ for 0, 1, 2, 4, and 8 h. We measured the levels of oxidative stress and analyzed the changes in molecules related to lipid metabolism. The levels of oxidative stress were found to be significantly elevated in Huh-7 hepatocellular carcinoma cells after O_3_ exposure. Moreover, the expression levels of intracellular lipid synthases, including SREBP1, FASN, SCD1, and ACC1, were enhanced. Lipolytic enzymes, including ATGL and HSL, and the mitochondrial fatty acid oxidase, CPT1α, were inhibited after O_3_ exposure. In addition, short O_3_ exposure enhanced the expression of the intracellular peroxisomal fatty acid β-oxidase, ACOX1; however, its expression decreased adaptively with longer exposure times. Overall, O_3_ exposure induces an increase in intracellular oxidative stress and disrupts the normal metabolism of lipids in hepatocytes, leading to intracellular lipid accumulation.

## Graphical abstract

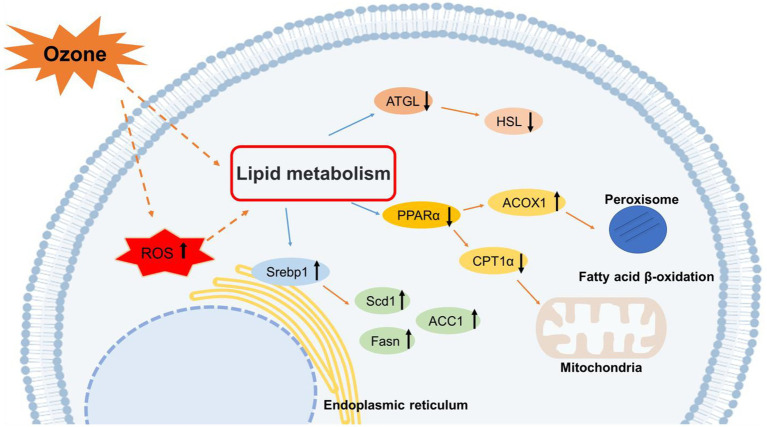

## 1. Introduction

Air pollution has significant adverse effects on human health. Based on previous studies, air pollution is associated with various diseases, including cardiovascular, respiratory, and metabolic diseases, and cancer (1–3). As a result, particulate-matter pollutants and their control measures have received increasing attention in recent years.

Currently, remarkable achievements have been made in PM_2.5_ control; however, ozone (O_3_) pollution has become a major environmental problem. O_3_ is a highly oxidizing substance with strong oxidizing properties that is present in large quantities in the environment. Absorbed O_3_ can cause oxidative damage in the body by generating ROS (4, 5). Acute and chronic ozone exposure increased the accumulation of superoxide and lipid peroxides in rats, leading to an increase in the oxidative stress levels (6). Previous studies have reported that acute ozone exposure can cause endocrine metabolic dysfunction in humans (7). However, the possible contribution of O_3_ to metabolic disorders and intrahepatic lipid metabolism is yet to be systematically investigated.

Lipid metabolism is an important part of human life and is closely associated with cancer, metabolic diseases, and cardiovascular diseases (8). In a study investigating the effects of long-term ozone exposure on human lipids, serum levels of triglycerides (TG) and high-density lipoprotein cholesterol (HDL-C) were found to increase, whereas those of total cholesterol (TC) and low-density lipoprotein cholesterol (LDL-C) decreased with increasing O_3_ concentrations (9). Other studies have found that acute ozone exposure significantly increases the blood serum levels of acylglycerol, glycerol, medium-chain free fatty acids, long-chain free fatty acids, and lysophosphatidic acid, and comprehensively alters peripheral lipid generation (10). Kim et al. (11) found that short-term ozone exposure is associated with increased serum triglyceride and VLDL-C levels and decreased HDL-C levels in humans. By exposing rats chronically to 1.0 ppm ozone, Miller et al. (12) found significant increases in HDL and total cholesterol levels, no significant change in triglyceride and free fatty acid levels, and significant decreases in the levels of these metrics during the recovery period after ozone exposure. Previous studies have revealed an increase in total serum cholesterol levels in older adult individuals due to increasing ozone exposure (13). Most studies investigated the effect of ozone exposure on serum lipid levels, whereas only few studies determined the effect of ozone exposure on human hepatic lipid metabolism factors.

The effects of ozone exposure on hepatic lipid metabolism are unknown. In fact, most studies focused on inflammatory processes in the lungs and metabolic diseases caused by chronic or acute ozone exposure. Although the lungs are the first target organ of O_3_ exposure, other tissues may be affected due to O_3_ transport through the blood, an important circulating body fluid. However, no studies have reported the effects of ozone exposure on hepatic lipid metabolism in the liver, an important organ that dominates lipid metabolic processes. Therefore, the aim of this study was to determine the effects of acute ozone exposure on hepatic lipid metabolic function, detect the levels of oxidative stress and the expression levels of related lipid metabolic factors in the liver using a cellular exposure assay, and explore the possible molecular mechanisms of O_3_-induced liver injury.

## 2. Materials and methods

### 2.1. Materials and reagents

Huh-7 cells were purchased from BNCC (Henan, China). CM-H2DCFDA was purchased from MCE (Shanghai, China). The triglyceride assay kit was purchased from Applygen (Beijing, China). Dulbecco’s modified Eagle medium (DMEM), trypan blue staining solution, and all other drugs and reagents were purchased from Invitrogen (Carlsbad, CA, United States).

### 2.2. Cell culture and O_3_ exposure

In this study, the human hepatocellular carcinoma cell line, Huh-7, was used to establish a cell model. Huh-7 cells are easy to culture and are widely used in contaminant exposure studies. The Huh-7 cell line was purchased from Henan Industrial Microbial Engineering Technology Research Center. Huh-7 cells were cultured in DMEM (Gibco BRL, Grand Island, NY, United States) containing 10% calf serum (N-10) in a 5% CO_2_ incubator at 37°C. The cells were seeded in a 6-well plate at 8 × 10^5^ cells per well, and incubated for 24 h. Toward the end of cell culture, the concentration of O_3_ in the exposure chamber was adjusted to 200 ppb, and four parallels of the same cell concentration were established for each group and placed in the cell exposure chamber for 0, 1, 2, 4, and 8 h. After O_3_ exposure, the cells were collected and frozen at −80°C for analyses. All cell experiments were performed three times, and the results are presented as mean values ± standard deviation.

### 2.3. Cell integrity assay

Trypan blue staining solution (Carlsbad, CA, United States) is a cell-reactive dye commonly used to determine the integrity of cell membranes and detect cell viability. Normal living cells with intact cell membranes can reject trypan blue, preventing its entry into the cell. In contrast, cells with inactive or incomplete cell membranes have increased permeability and are stained blue by the dye. Imaging was performed using a laser-scanning confocal microscope (Thermo Fisher Scientific).

### 2.4. Cell viability assay

A CCK-8 kit was used for therapid and sensitive detection of cell proliferation and cytotoxicity. In the presence of electronically coupled reagents, WST-8 can be reduced by mitochondrial dehydrogenases to produce a highly water-soluble orange-yellow methanogenic product; the color shade of this product is proportional to cell proliferation and inversely proportional to cytotoxicity. Moreover, for the same cells, the shade of color and the number of cells are linearly related. The optical density (OD) was measured at 450 nm using an enzyme standard meter, which indirectly reflects the number of living cells. Spectrophotometric measurements were performed using a MiniMax multifunctional enzyme analyzer (Molecular Devices, United States).

### 2.5. Measurement of intracellular ROS content

The cells were treated with 10 μM CM-H2DCFDA in the dark at 37°C for 30 min, and the excess probe was removed via washing. The samples were analyzed using a Fluorescence Microscope (Thermo Fisher Scientific, United States).

### 2.6. Measurement of intracellular triglyceride content

The Triglycerides (TG) concentration in Huh-7 cells was determined using a Triglyceride assay kit (Beijing, China), according to the manufacturer’s instructions.

### 2.7. Total RNA extraction and qPCR

Total RNA was extracted from the cultured cells using TRIzol reagent (Invitrogen, Carlsbad, CA, United States), according to the manufacturer’s instructions. RNA was quantified using a NanoDrop 2000 spectrometer (Thermo Fisher Scientific, United States). Reverse transcription was performed using 2 mg RNA at 42°C for 50 min with a mixture containing 200 U SuperScript II reverse transcriptase enzyme, 125 ng random primers, and 0.5 mM dNTP Mix (Invitrogen, United States).

Real-time PCR validation was conducted using the Maxima® SYBR® Green qPCR Master Mix kit (Takara Bio, Dalian, China), according to the manufacturer’s instructions, in an ABI Prism 7500 Sequence Detection System 288 (Applied Biosystems Inc.). The following cycling condition were employed: 95°C for 10 min; 95°C for 30 s, 60°C for 1 min, and 72°C for 1 min; 40 cycles. *GAPDH* was used as the standardized internal control. The primer sequences are listed in Table 1. Each sample was repeated in triplicate and the fold change in gene expression was calculated according to the 2^-ΔΔCt^ method.

**Table 1:** Sequence of primers of used for qPCR.

Gene	Sequence of primer (5′ → 3′)		Amplified fragment size
	Forward	Reverse		Huh-7 cell	
*ACOX1*	TGCCTATGCCTTCCAGTT	TGCTTCAATGCCAGTGTT	163
*CPT1a*	AAGTTGGCGTCTGAGAAG	GGTCTGGCTTGTTGATAATC	168
*SREBP1*	CACCAGCGTCTACCATAG	AGAGAAGCACCAAGGAGA	140
*FASN*	GTGGTCTTCTCCTCTGTGA	GTTGGTGCTCATCGTCTC	180
*ACC1*	CTTCCTGCTCATACACTTCT	GTATAACTGCTGCCATCATAG	191
*SCD1*	GGAGGAGATAAGTTGGAGAC	GTAGCAGAGACATAAGGATGA	157
*ATGL*	CGGCGAGAATGTCATTATATC	CATAGAGTGGCAGGTTGTC	161
*HSL*	GGAAGTGCTATCGTCTCTG	GATGTTCAATGCTCCACTG	193
*PPARα*	ATCGGCGAGGATAGTTCT	GAGCTTCAGCCCAGGGTC	180
*GAPDH*	GGCTCTCCAGAACATCATC	CCTGCTTCACCACCTTCT	191

### 2.8. Enzyme-linked immunosorbent assay

The cells were thoroughly mixed with lysate NP-40 (Beyotime, Shanghai, China) and centrifuged. The supernatant was then collected and used for protein expression analysis. Enzyme-linked immunosorbent assay (ELISA) was performed to determine the expression levels of target proteins in Huh-7 cells. The ELISA kits were purchased from Beijing Qisong Biotechnology (PPAR-α: QS43373; Srebp-1: QS46773; ACOX1: QS43381; ATGL: QS48838; ACC1: QS46723; CPT1α: QS46824; FASN: QS46882; SOD1: QS46774; HSL: QS46881; CYP1A1: QS43372; and SCD1: QS46897). All reagents, samples, and standards were prepared according to the manufacturer’s instructions.

### 2.9. Statistical analysis

The results are presented as mean ± standard deviation. Significant differences between the groups were analyzed using one-way ANOVA. All statistical analyses were performed using GraphPad Prism 8.0. Differences were considered statistically significant at *p* < 0.05.”

## 3. Results

### 3.1. Effect of O_3_ exposure on Huh-7 cell viability

**Figure 1:**
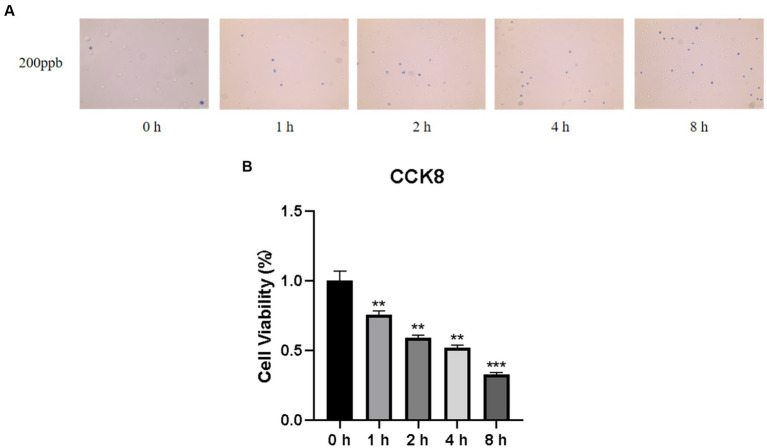
Results of cell viability assay of Huh-7 cells after O_3_ exposure. **(A)** Results of *Trypan blue staining* in the background with different time gradients of ozone exposure. **(B)** Huh-7 cells were treated with control or 200 ppb O_3_ for 1, 2, 4, and 8 h, respectively, and cell viability was measured using CCK-8 method. *p < 0.05, **p < 0.01,***p < 0.001 vs. control group.

Cell viability is a valid indicator of cell damage and can be determined via cell staining and cell counting. To detect the cytotoxicity of O_3_, we used trypan blue staining and CCK8 cell counting to determine the survival rate of Huh-7 cells after different time gradients of O_3_ exposure. As shown in Figure 1A, the number of dead cells (stained blue) gradually increased as the O_3_ exposure time increased. Cell survival declined continuously with increasing O_3_ exposure time and cell activity was significantly downregulated in the exposed group compared to that in the control group (Figure 1B).

### 3.2. Effect of O_3_ exposure on intracellular ROS

**Figure 2:**
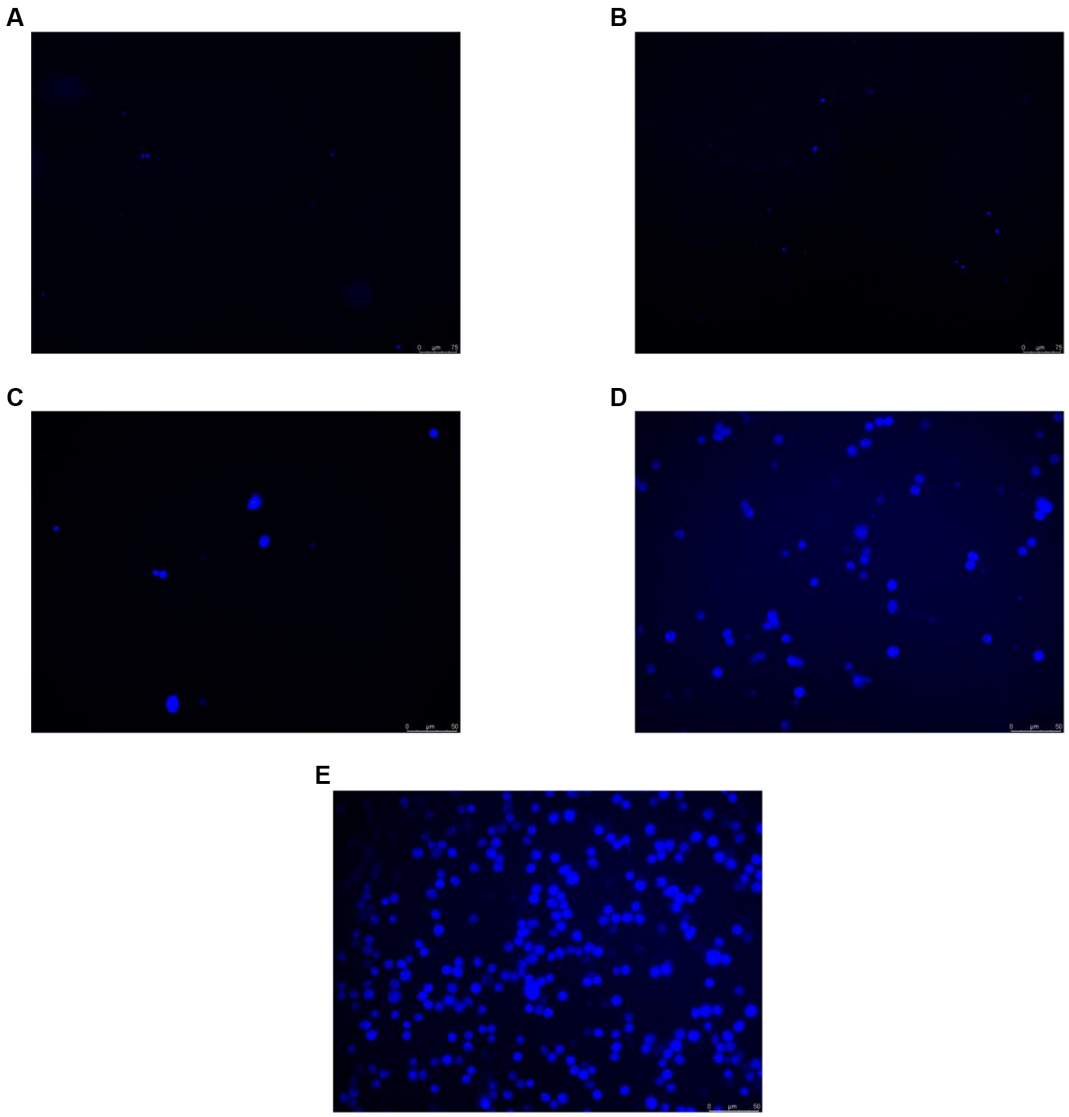
CM-H2DCFDA staining results of Huh-7 cells after 200 ppb O_3_ exposure. **(A)** Intracellular ROS content after 200 ppb O_3_ exposure for 0 h. **(B)** Intracellular ROS content after 200 ppb O_3_ exposure for 1 h. **(C)** Intracellular ROS content after 200 ppb O_3_ exposure for 2 h. **(D)** Intracellular ROS content after 200 ppb O_3_ exposure for 4 h. **(E)** Intracellular ROS content after 200 ppb O_3_ exposure for 8 h.

To verify whether O_3_ exposure caused changes in intracellular ROS levels, Huh-7 cells were treated with 200 ppb O_3_ for 0, 1, 2, 4, or 8 h. The CM-H2DCFDA assay revealed that intracellular ROS levels increased with increasing O_3_ exposure time (Figure 2).

### 3.3. Effects of O_3_ exposure on the expression of CYP1A1 and SOD1 in Huh-7 cells

**Figure 3:**
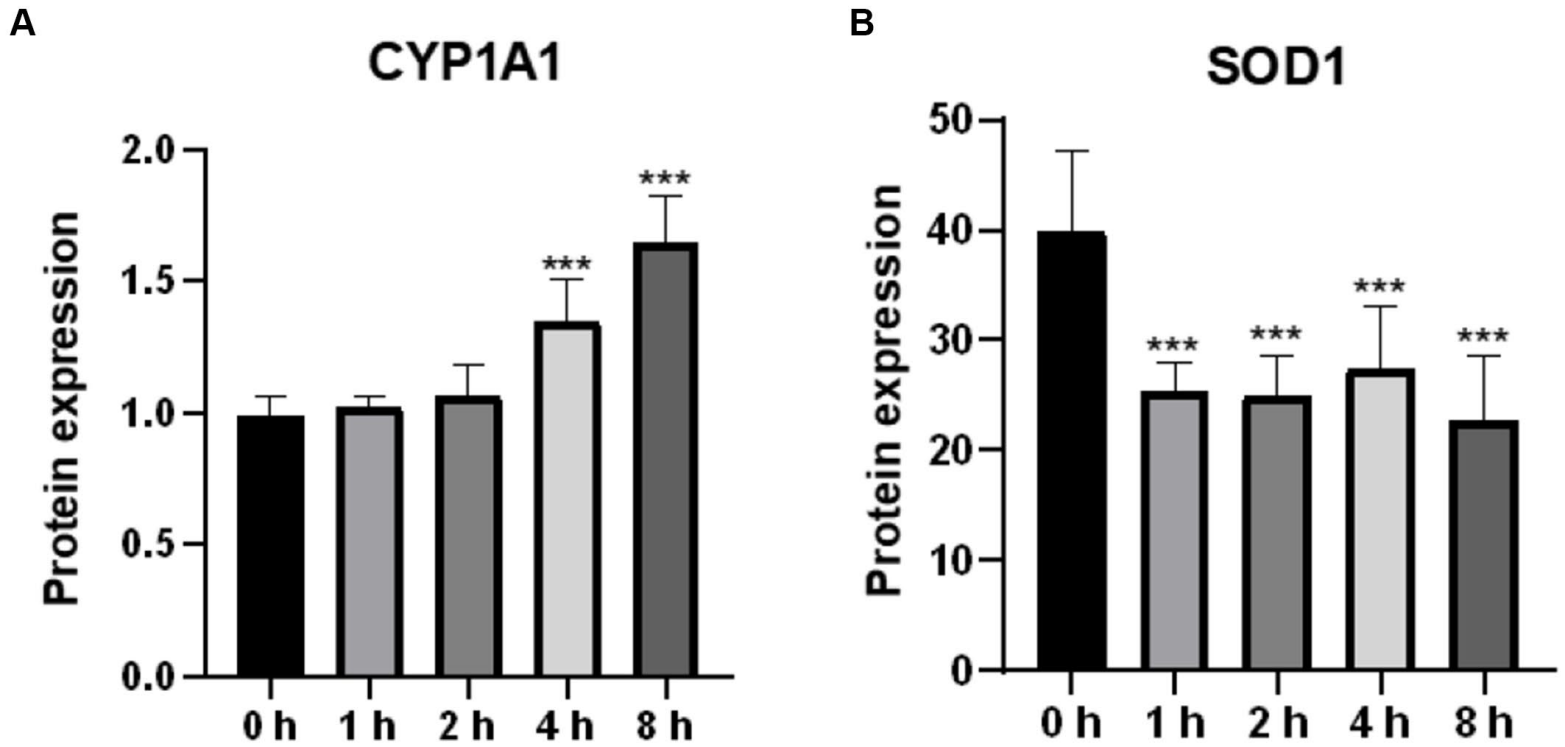
Effect of O_3_ exposure on protein expression of oxidative stress-related enzymes. Protein expression levels of CYP1A1 **(A)** and SOD1 **(B)** in Huh-07 cells at different time gradients (0, 1, 2, 4, and 8 h) after O_3_ exposure are expressed as mean ± SE (*n* = 3 cells/group). *p < 0.05, **p < 0.01,***p < 0.001 vs. control group.

Oxidative stress is caused by an imbalance between the oxidation and antioxidant levels in the body. In the present study, we examined the protein levels of CYP1A1 and SOD1, which are associated with reactive oxygen species production and antioxidant activity, respectively. As shown in Figure 3A, CYP1A1 expression levels were significantly higher in cells treated with O_3_ for 4 and 8 h than in the corresponding control cells. We also examined the expression of SOD1. Based on our findings, the expression level of SOD1 in cells was significantly reduced after O_3_ exposure (Figure 3B).

### 3.4. Effect of O_3_ exposure on intracellular triglyceride content

**Figure 4:**
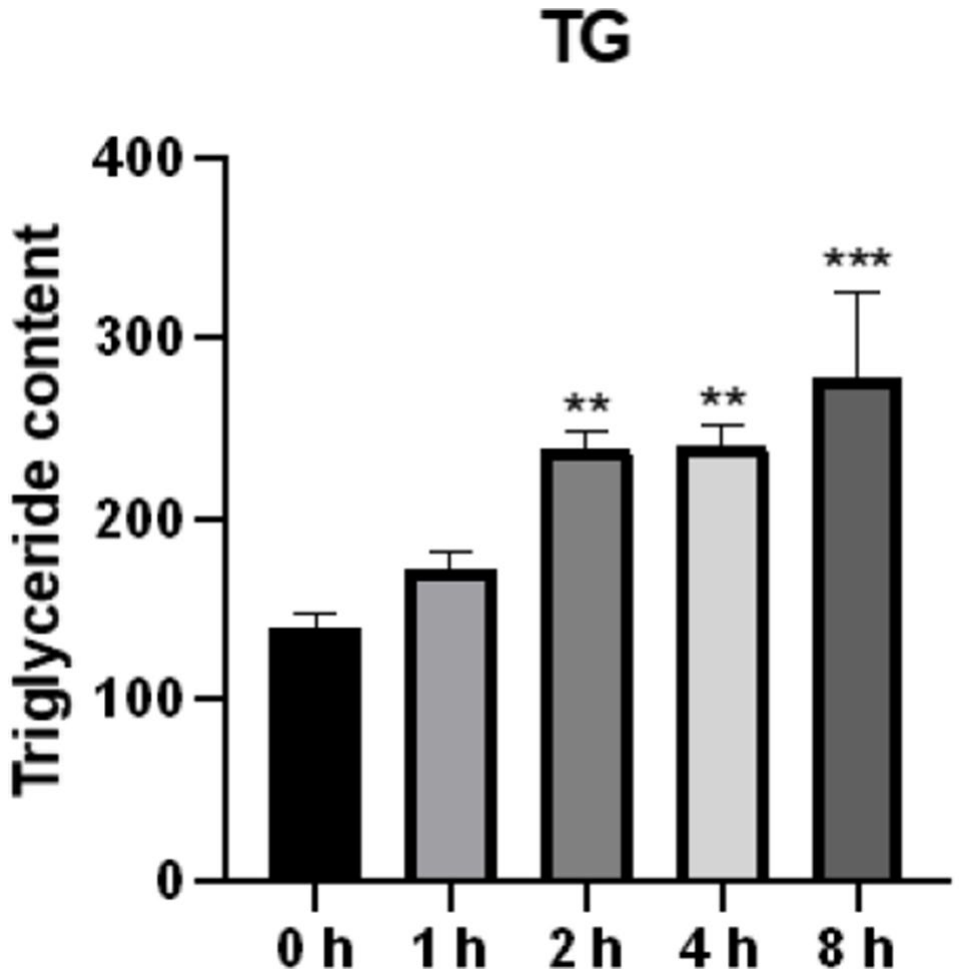
Effect of O_3_ exposure on intracellular triglyceride content. The level of TG in Huh-07 cells at different time gradients (0, 1, 2, 4, and 8 h) after O_3_ exposure are expressed as mean ± SE (*n* = 3 cells/group). *p < 0.05, **p < 0.01,***p < 0.001 vs. control group.

A high TG level is one of the main characteristics of lipid accumulation. In the present study, intracellular triglyceride levels were found to be significantly elevated after O_3_ exposure (Figure 4).

### 3.5. Effects of O_3_ exposure on the expression of genes involved in lipid synthesis

**Figure 5:**
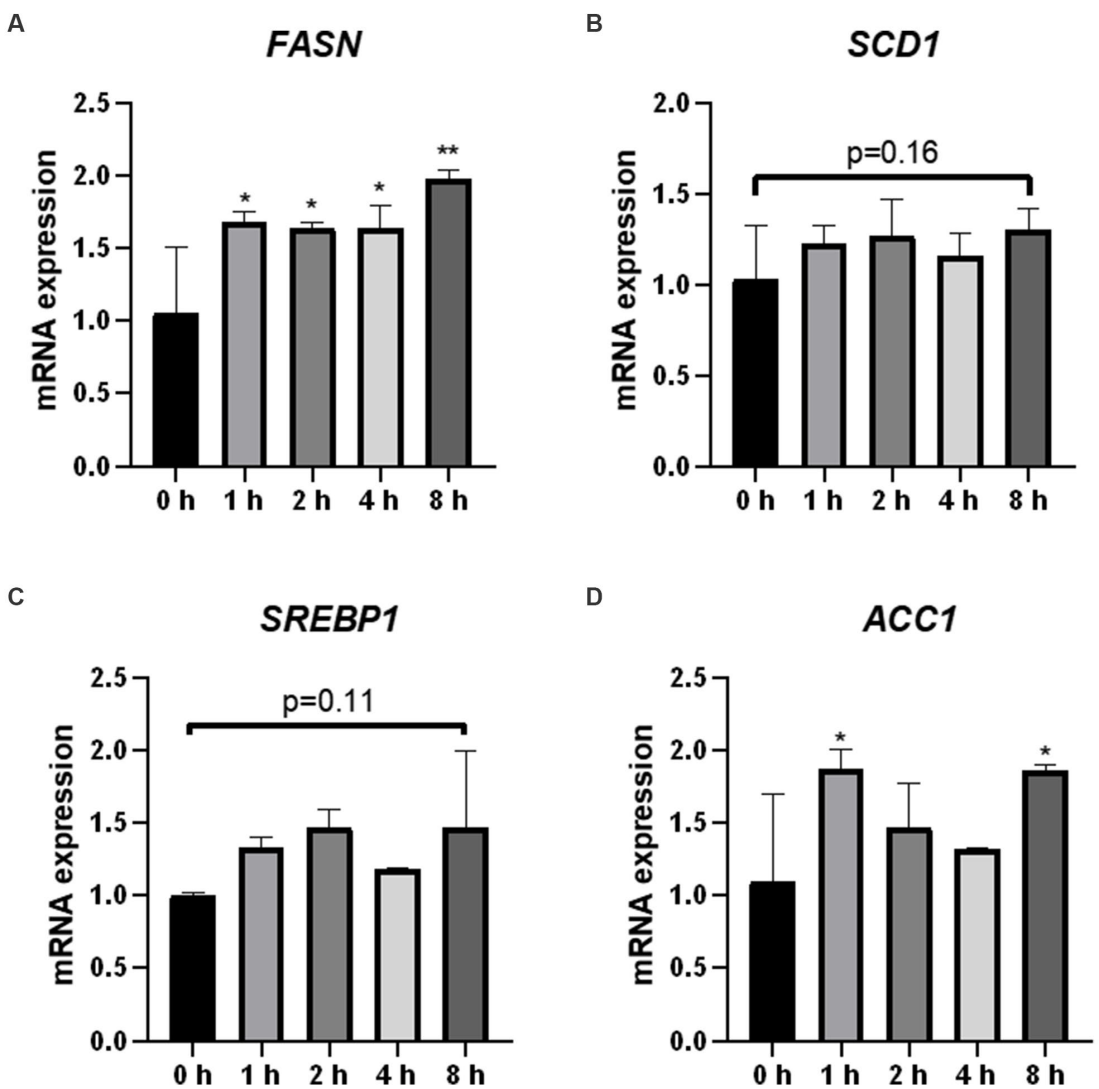
Effect of O_3_ exposure on mRNA expression of lipid synthesis-related enzymes. mRNA expression levels of FASN **(A)**, SCD1 **(B)**, SREBP1 **(C)**, and ACC1 **(D)** in Huh-7 cells at different time gradients (0, 1, 2, 4, and 8 h) after O_3_ exposure are expressed as mean ± SE (*n* = 3 cells/group). ^*^p < 0.05, ^**^p < 0.01 vs. control group.

To demonstrate the effects of O_3_ exposure on lipid synthesis in Huh-7 cells, we evaluated the expression of four genes related to lipid synthesis. The mRNA expression of *FASN* was significantly upregulated after ozone exposure (Figure 5A). In addition, the mRNA expression of *ACC1* was significantly upregulated after 1 and 8 h of O_3_ exposure (Figure 5D). Although there were no significant changes in the mRNA expression of *SCD1* and *SREBP1* in the cells, their mRNA expression levels were upregulated after 8 h of O_3_ exposure (*p* = 0.16, *p* = 0.11; Figures 5B,C).

### 3.6. Effects of O_3_ exposure on the expression of genes involved in lipid catabolism

**Figure 6:**
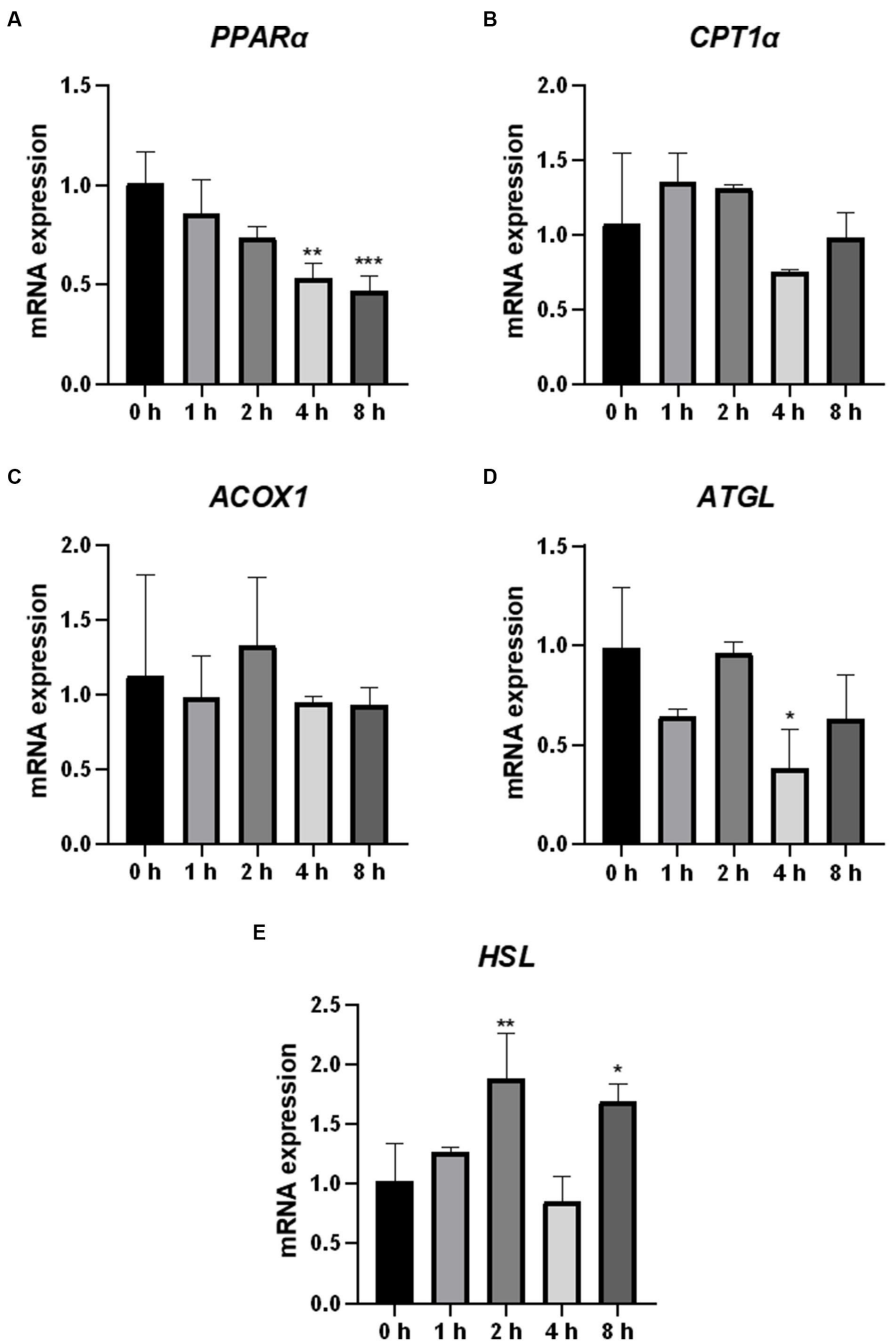
Effect of O_3_ exposure on mRNA expression of genes encoding key enzymes or regulators involved in fatty acid oxidation and lipolysis in Huh-7 cells. mRNA expression levels of PPARα **(A)**, CPT1*α* **(B)**, ACOX1 **(C)**, ATGL **(D)**, and HSL **(E)** in Huh-7 cells at different time gradients (0, 1, 2, 4, and 8 h) after O_3_ exposure are expressed as mean ± SE (*n* = 3 cells/group). *p < 0.05, **p < 0.01,***p < 0.001 vs. control group.

Fatty acids are the core components of most lipids. We analyzed the mRNA expression of the genes (*PPARα*, *CPT1*α, *ACOX1*, *ATGL*, and *HSL*) encoding the key enzymes and regulators involved in fatty acid oxidation and lipolysis. *HSL* mRNA expression was significantly upregulated in cells 2 h after O_3_ exposure (Figure 6E). The mRNA expression levels of *PPARα* and *ATGL* were significantly downregulated in the cells after 4 h of O_3_ exposure (Figures 6A,D). After 8 h of ozone exposure, the mRNA expression level of *PPARα* was significantly downregulated, while that of *HSL* was significantly upregulated (Figures 6A,E). No significant changes in the mRNA expression levels of *CPT1*α and *ACOX1* were found in cells after O_3_ exposure (Figures 6B,C).

### 3.7. Effect of O_3_ exposure on protein expression related to lipid metabolism in Huh-7 cells

**Figure 7:**
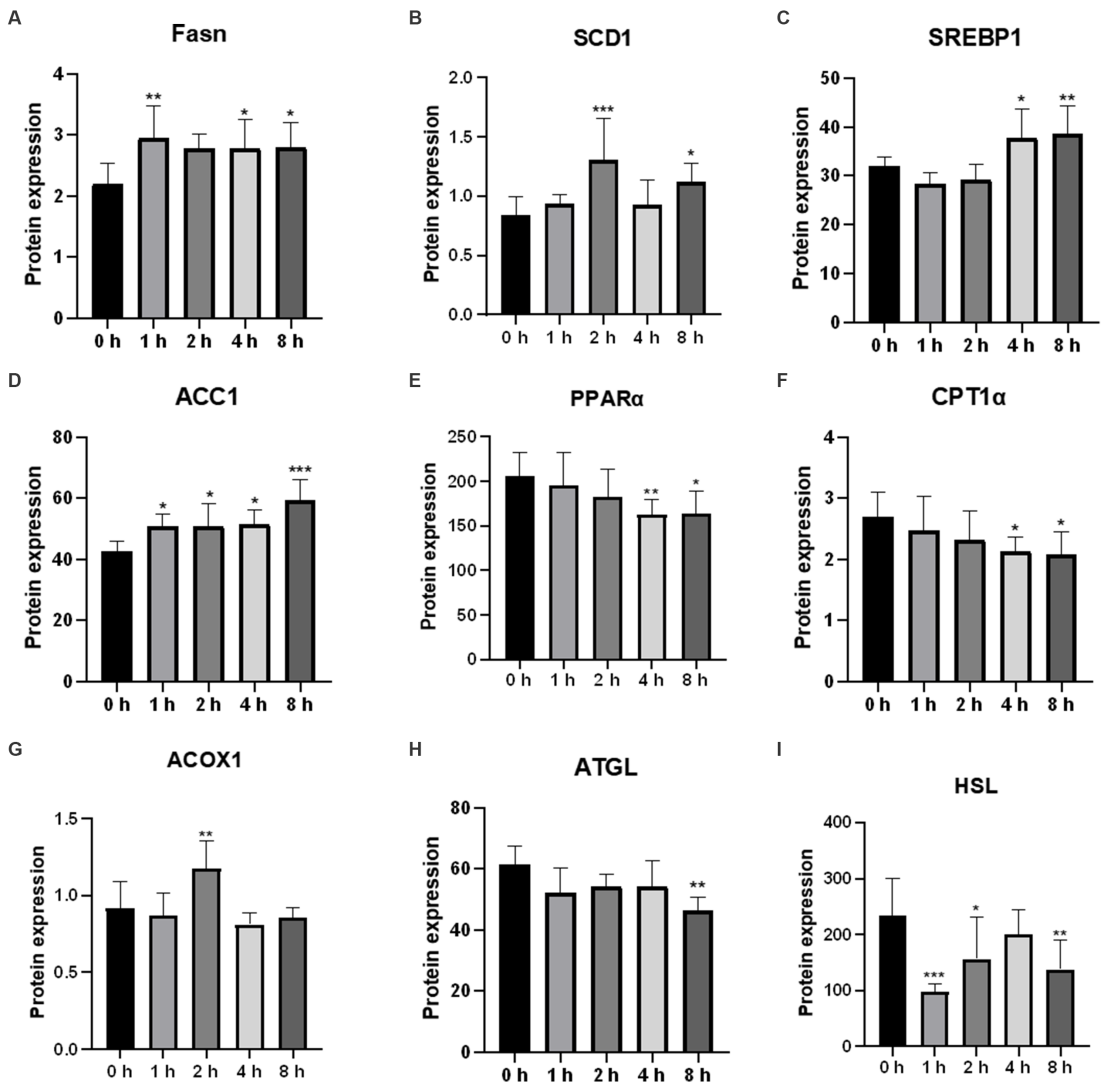
Effects of O_3_ exposure on the protein expression of lipid metabolism-related genes in Huh-7 cells at different time gradients (0, 1, 2, 4, and 8 h). **(A)** Protein expression of FASN; **(B)** Protein expression of SCD1; **(C)** Protein expression of SREBP1; **(D)** Protein expression of ACC1; **(E)** Protein expression of PPARα; **(F)** Protein expression of CPT1*α*; **(G)** Protein expression of ACOX1; **(H)** Protein expression of ATGL; **(I)** Protein expression of HSL. Data were expressed as mean ± SE (*n* = 3 cells/group). *p < 0.05, **p < 0.01,***p < 0.001 vs. control group.

To further confirm the effect of O_3_ exposure on the expression of genes involved in lipid metabolism, ELISA was performed to validate the expression levels of proteins. After 1 h of exposure to O_3_, the protein expression levels of FASN and ACC1 were significantly upregulated, whereas that of HSL was significantly downregulated in Huh-7 cells (Figures 7A,D,I). After 2 h of exposure to O_3_, the protein expression levels of SCD1, ACOX1, and ACC1 were significantly upregulated, whereas that of HSL was significantly downregulated (Figures 7B,D,G,I). After 4 h of exposure to O_3_, the protein expression levels of FASN, SREBP1, and ACC1 were significantly upregulated, while those of PPARα and CPT1α were significantly downregulated (Figures 7A,C–E,I). After 8 h of exposure to O_3_, the protein expression levels of FASN, SCD1, SREBP1, and ACC1 were significantly downregulated, while those of PPARα, CPT1α, ATGL, and HSL were significantly downregulated (Figures 7A–F,H,I).

## 4. Discussion

Many studies have shown that O_3_ pollution adversely affects human health (14); however, evidence of its impact on the liver is limited (15). According to previous studies, disrupted lipid metabolism can lead to liver fibrosis (16). Therefore, to verify the effect of O_3_ on the liver, we exposed Huh-7 hepatocellular carcinoma cells to different ozone time gradients and measured the changes in oxidative stress and lipid metabolic factors. Cell viability was found to decrease in the exposed group compared to the control group, and intracellular ROS and TG contents increased with increasing O_3_ exposure time. The expression levels of lipid synthesis-related genes and their downstream proteins were upregulated, whereas those of fatty acid oxidation and lipid catabolic genes and their downstream proteins were downregulated. These results suggest that 200 ppb O_3_ is cytotoxic and O_3_ exposure may induce oxidative stress and impair intracellular lipid metabolism in Huh-07 hepatocellular carcinoma cells, resulting in intracellular lipid accumulation.

Based on animal and human studies, oxidative stress plays an important role in the pathogenesis of nonalcoholic fatty liver disease (NAFLD) (17–19). O_3_, a strong oxidant, is directly related to oxidative stress. *In vitro* studies revealed that O_3_ induces the release of ROS from animal respiratory epithelial cells and causes the dysregulation of oxidative stress levels in the organism (20). Such finding is consistent with our results, which showed an increase in intracellular ROS levels with increasing O_3_ exposure time. We also determined the protein levels of intracellular CYP1A1 and SOD1, which are associated with reactive oxygen species production and antioxidant activity, respectively. An increase in intracellular CYP1A1 levels or a decrease in SOD1 levels can lead to intracellular oxidative stress (21). The protein levels of intracellular CYP1A1 were significantly upregulated after O_3_ exposure, whereas those of SOD1 were significantly downregulated. These results indicate that O_3_ exposure leads to elevated levels of oxidative stress in Huh-7 hepatocellular carcinoma cells.

Disturbance of hepatic lipid metabolism is a symptom of NAFLD exacerbation. Inflammatory activity induces collagen synthesis in stellate cells and promotes liver fibrosis, further contributing to the exacerbation of NAFLD (22, 23). However, the exact cause of abnormalities in hepatic lipid metabolism caused by O_3_ exposure *in vivo* is unknown. Therefore, we determined the mRNA and protein expression levels of genes involved in lipid anabolism in Huh-7 cells exposed to O_3_. Serbp1 is an intracellular cholesterol sensor located in the endoplasmic reticulum, which provides feedback for cholesterol regulation. SREBP1 is involved in the regulation of FASN, ACC1, and SCD1 expression (24, 25). FASN is a key enzyme in the *de novo* synthesis of fatty acids (26). ACC1 is the first rate-limiting enzyme in the *de novo* lipogenesis (DNL) process, which plays an important role in fatty acid synthesis (27). Scd-1 is a rate-limiting enzyme that catalyzes the synthesis of monosaturated fatty lipids (28). Our results revealed that the mRNA expression levels of FASN and ACC1 were significantly elevated in Huh-7 cells exposed to 200 ppbO_3_. Although the upregulation trend for the mRNA expression of SREBP1 and SCD1 was not obvious, their protein expression levels were significantly higher in the exposed group, which was consistent with the general trend of changes in gene levels. These findings suggest that O_3_ exposure induces hepatic lipid synthesis.

Lipolysis, the sequential hydrolysis of triacylglycerols stored in cellular lipid droplets, plays an important role in lipid metabolism (29). ATGL and HSL lipases are considered the main regulators of lipolysis (30). ATGL is the first step in the catalysis of lipolysis, converting TGs to diacylglycerols and FFAs. HSL hydrolyzes diacylglycerols to monoacylglycerols and FFAs, which are mainly regulated by ATGL (31, 32). Fatty acids formed by the hydrolysis of triglycerides are mainly metabolized via β-oxidation, which primarily occurs in the mitochondria and peroxisomes (33). Several key enzymes in these fatty acid oxidation pathways are regulated by PPARα. Mitochondrial oxidation is regulated by CPT1α. ACOX1 is a rate-limiting enzyme that catalyzes the first step of peroxisomal fatty acid oxidation (34). In the present study, the mRNA expression levels of HSL were upregulated, and those of ATGL were downregulated after O_3_ exposure. The mRNA expression levels of PPARα were significantly downregulated after 4 and 8 h of O_3_ exposure, while those of CPT1α did not significantly change. The mRNA expression levels of ACOX1 were upregulated after 2 h of O_3_ exposure. In addition, the protein expression levels of ATGL and HSL were downregulated after O_3_ exposure compared to those of the control. The protein expression levels of CPT1α were significantly downregulated after 4 and 8 h of O_3_ exposure. The protein expression levels of PPARα and ACOX1 significantly correlated with gene expression. Regarding the differential expression of intracellular HSL genes and proteins, we speculate that the regulation of this factor by O_3_ mainly occurs at the protein level. Based on our findings, O_3_ exposure downregulates lipolysis and mitochondrial fatty acid-oxidation in Huh-7 cells, whereas short O_3_ exposure upregulates intracellular peroxisomal fatty acid-oxidation. These effects depend on the duration of O_3_ exposure.

Nonalcoholic fatty liver disease has become one of the most common liver diseases and is responsible for a significant number of liver disease cases (35, 36). According to previous studies, ozone exposure increases the levels of lipid metabolites in humans (7). Moreover, disruption of hepatic lipid metabolism leads to accumulation of liver fat, which further causes liver inflammation leading to the development of NAFLD. Our results suggest that O_3_ exposure may lead to lipid accumulation by regulating the expression of lipid metabolism-related factors, resulting in elevated levels of lipid synthesis and decreased levels of lipolysis and intramitochondrial fatty acid-oxidation.

## 5. Conclusion

In summary, our findings suggest that O_3_ exposure causes dysfunctional lipid metabolism in hepatocytes, which leads to the accumulation of intracellular lipids. The specific regulatory mechanism between the intrahepatic inflammatory response and lipid metabolism induced by O_3_ exposure should be investigated in a future study.

## Data availability statement

The original contributions presented in the study are included in the article/supplementary material; further inquiries can be directed to the corresponding authors.

## Author contributions

JP: conceptualization, methodology, formal analysis, investigation, writing—original draft, visualization, and review and editing. SW: conceptualization, validation, investigation, and writing—review and editing. YW: conceptualization, methodology, formal analysis, investigation—original draft, and visualization. WY: software, investigation, and resources. ZY: conception, verification, investigation—review and editing. SG: conceptualization, methodology, formal analysis, investigation, writing—original draft, and visualization. All authors contributed to the article and approved the submitted version.

## Funding

This study was supported by Inner Mongolia Natural Science Foundation (No. 2019MS03077); the fifth batch of “Grassland Talents” Industry Innovation and Entrepreneurship Talent Team in Inner Mongolia Autonomous Region: “Beef Cattle Scientific Research and Beef Cattle Industry Innovation Talent Team” special project; Inner Mongolia major special project “Horqin Beef Breed Breeding,” subproject “Horqin Beef Cattle Physiological and Biochemical Index Test Analysis”; and Inner Mongolia Autonomous Region Science and Technology Project “Horqin Beef Cattle New Breeding Integrated Technology Research and Demonstration Promotion,” subproject “Inheritance of Beef Quality Traits Mechanism technology research” (KJXM2020002-05).

## Conflict of interest

The authors declare that the research was conducted in the absence of any commercial or financial relationships that could be construed as a potential conflict of interest.

## Publisher’s note

All claims expressed in this article are solely those of the authors and do not necessarily represent those of their affiliated organizations, or those of the publisher, the editors and the reviewers. Any product that may be evaluated in this article, or claim that may be made by its manufacturer, is not guaranteed or endorsed by the publisher.
